# Resolving the Molecular Steps in Clostridial Neurotoxin Light Chain Translocation

**DOI:** 10.33696/Neurol.1.020

**Published:** 2020

**Authors:** Madison Zuverink, Joseph T. Barbieri

**Affiliations:** 1 Dalhousie University, Department of Biochemistry and Molecular Biology, Halifax, Nova Scotia, Canada; 2 Medical College of Wisconsin, 8701 Watertown Plank Road, BSB2 Rm. 2830, Microbiology and Immunology, Milwaukee, WI 53226, USA

## Abstract

The clostridial neurotoxins (CNTs), botulinum toxin and tetanus toxin, are the most toxic proteins for humans. Neurotoxicity is based upon the specificity of the CNTs for neural host receptors and substrates. CNTs are organized into three domains, a Light Chain (LC) that is a metalloprotease and a Heavy Chain (HC) that has two domains, an N-terminal LC translocation domain (HCN) and a C-terminal receptor binding domain (HCC). While catalysis and receptor binding functions of the CNTs have been developed, our understanding of LC translocation is limited. This is due to the intrinsic complexity of the translocation process and limited tools to assess the step-by-step events in LC translocation. Recently, we developed a novel, cell-based TT-reporter to measure LC translocation as the translocation of a β-lactamase reporter across a vesicle membrane in neurons. Using this approach, we identified a role for a *cis*-Loop, located within the HCN, in LC translocation. In this commentary, we describe our current understanding of how CNTs mediate LC translocation and place the role of the *cis*-Loop in the LC translocation process relative to other independent functions that have been implicated in LC translocation. Understanding the basis for LC translocation will enhance the use of CNTs in vaccine development and as human therapies.

## Introduction

### The clostridial neurotoxins, tetanus toxin and the botulinum toxins

Due to use as human vaccines and therapies, the clostridial neurotoxins (CNTs) have been subjected to decades of scientific investigation using biophysical, electrophysiological, and pharmacological approaches to establish mechanisms of toxin action. While progress has been made towards resolving the catalytic and host receptor mechanisms, and several conserved structures are implicated in the translocation process, the discrete steps in the translocation process remain cloak-and-dagger. Our recent study, “Tetanus toxin *cis*-Loop contributes to Light-Chain translocation,” by Zuverink et al. [1], identified a structurally conserved, charged loop (termed *cis*-Loop) that upon aliphatic mutation uncoupled LC translocation, but not pore-formation. In this commentary, we discuss potential functions of the *cis*-Loop by examining proteins containing similar secondary structural elements together with current translocation models. A deeper understanding of structure-function properties of LC translocation will facilitate identification of structural motifs in emerging bacterial toxins that can be targeted for the intracellular neutralization of toxin action.

### Clostridial neurotoxin pathology

**Figure 1:**
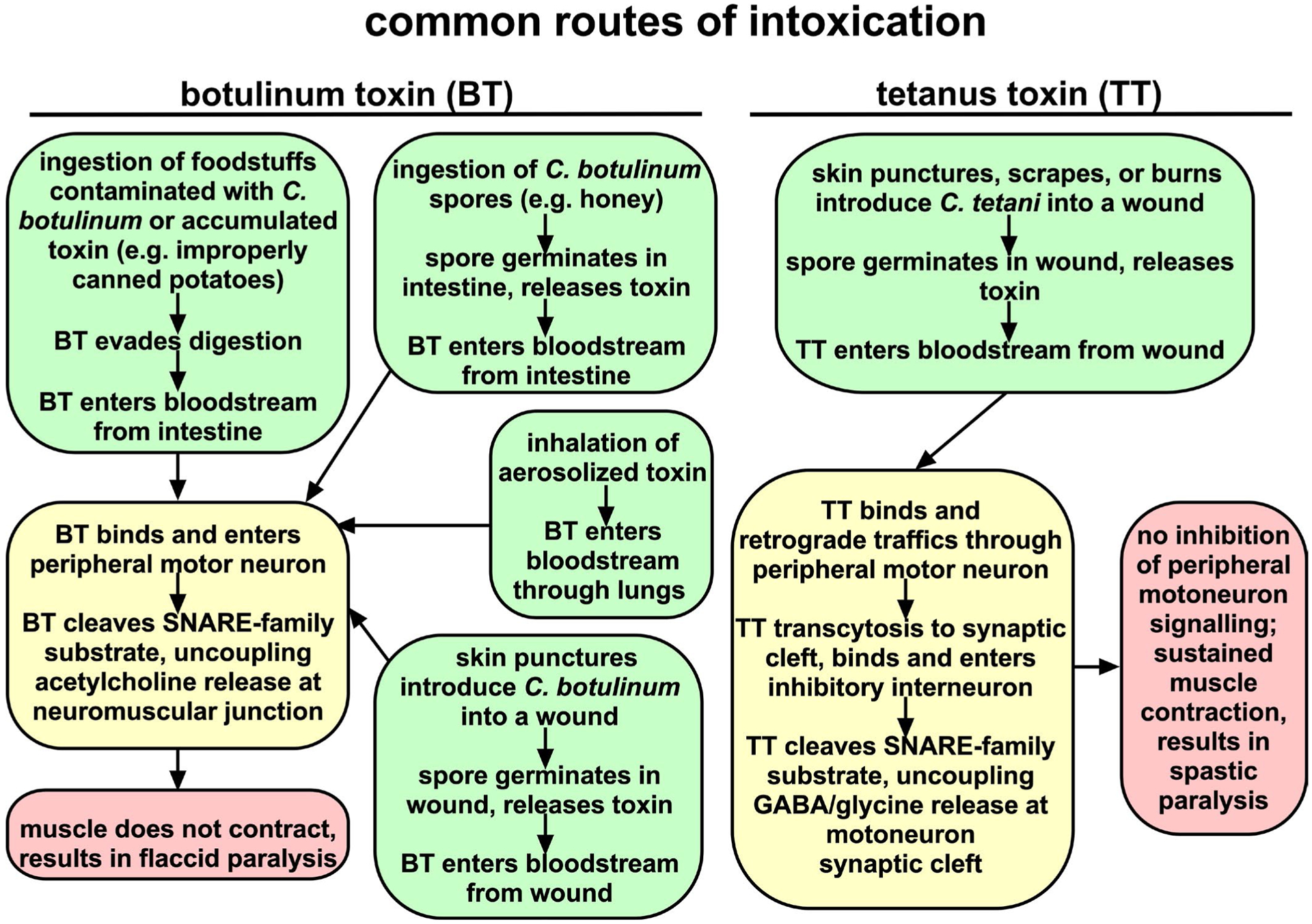
Common routes of intoxication. BT- and TT- routes of exposure are highlighted in yellow. Cellular mechanisms are highlighted in green and clinical presentation is highlighted in red. For BT, intestinal sporulation is observed predominantly in neonates.

**Figure 2:**
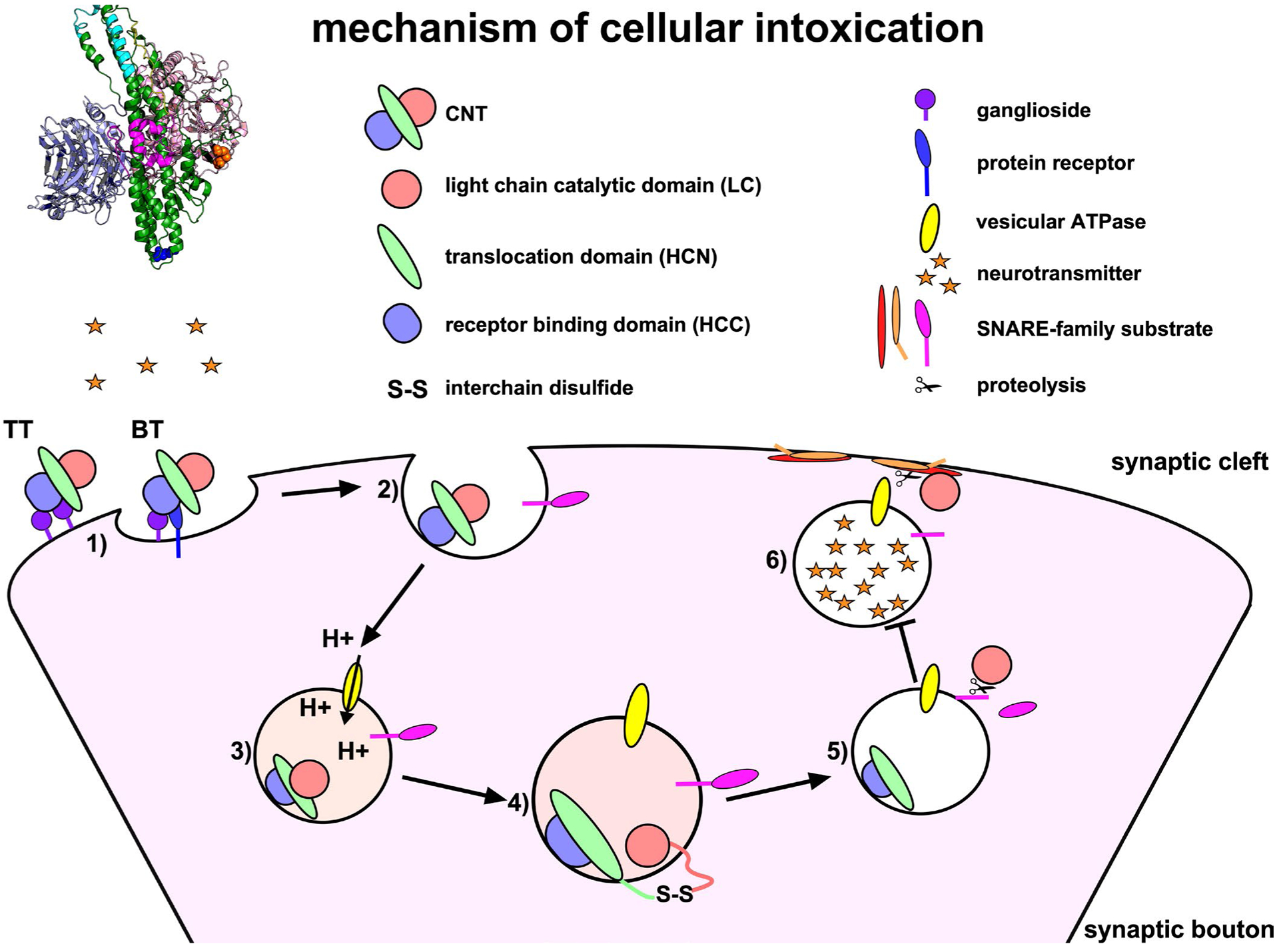
Mechanism of cellular intoxication. 1) At the target neuron, BT receptor binding domain (HCC) binds a synaptic vesicle protein and ganglioside receptor (peripheral motoneuron), while TT HCC binds dual gangliosides (inhibitory interneuron). 2) At peripheral motoneuron, BT enters a synaptic vesicle through receptor-mediated endocytosis, while at inhibitory interneuron, TT enters a yet to be defined vesicle. 3) As the vesicle matures, vesicular ATPases pump protons across the vesicular membrane, acidifying the lumen and inducing conformational changes in BT or TT. 4) BT and TT translocation domain (HCN) inserts into the vesicular membrane to facilitate LC translocation. 5) The interchain disulfide is reduced and the LC cleaves a SNARE-family substrate. 6) Proteolysis of the SNARE substrate inhibits SNARE-mediated vesicle fusion to the plasma membrane, uncoupling neurotransmitter release at the synaptic cleft.

*Clostridium tetani* and *C. botulinum* are ubiquitous soil organisms that produce CNTs. The CNTs include a highly conserved tetanus toxin (TT) and a multitude of serologically distinct botulinum neurotoxins (BT/A-G), which cause paralytic diseases, tetanus (spastic paralysis) and botulism (flaccid paralysis), respectively (Figure 1). Currently, most children receive vaccination to prevent tetanus, but there is no approved vaccine to prevent human botulism. Recently, bioinformatics data mining revealed unexpected BT homologues, including BT/X (*C. botulinum*), and non-clostridial BT/En (*Enterococcus faecium)*, BT/Pmp1 (*Paraclostridium bifermentans*) and BT-like toxin BT/Wo (*Weissella oryzae*) [2,3]. CNTs bind neuronal receptors and cleave SNARE (soluble n-ethylmaleimide-sensitive-factor attachment protein receptor) proteins (Figure 2). SNARE proteins contribute to the fusion of neurotransmitter containing vesicles to the neuronal plasma membrane [4]. BT cleaves SNARE proteins to prevent acetylcholine release by peripheral motor neurons at the neuromuscular junction, which results in botulism (Figure 1). TT retrograde traffics into the central nervous system and cleaves VAMP2, a SNARE protein, which prevents glycine release by inhibitory interneurons upstream of the peripheral motor neuron, which results in tetanus (Figure 1) [5].

### CNT organization and domain function

CNTs are ~150 kDa single chain proteins, which are cleaved into a di-chain protein by bacterial or host proteases. Dichain CNTs are covalently linked through an interchain disulfide composed of the N-terminal Light Chain (LC) and C-terminal Heavy Chain (HC). The di-chain proteins vary in tertiary conformation as open, closed, or pyramid structures [4]. LC is a zinc-metalloprotease that cleaves SNARE family proteins. The N-terminal domain of the HC encodes a LC translocation function (HCN), which delivers the LC into the host cytosol, and C-terminal domain encodes the host receptor binding function (HCC), which binds combinations of neuron-specific host receptors, including gangliosides, lipids, and synaptic vesicle proteins [4,6]. LC and HCC are soluble domains and can be expressed independently in *E. coli*, which has facilitated characterization of CNT-host receptor interactions at the cell membrane and intracellular targets.

## Conserved HCN Secondary Structural Elements Implicated in LC Translocation

**Figure 3:**
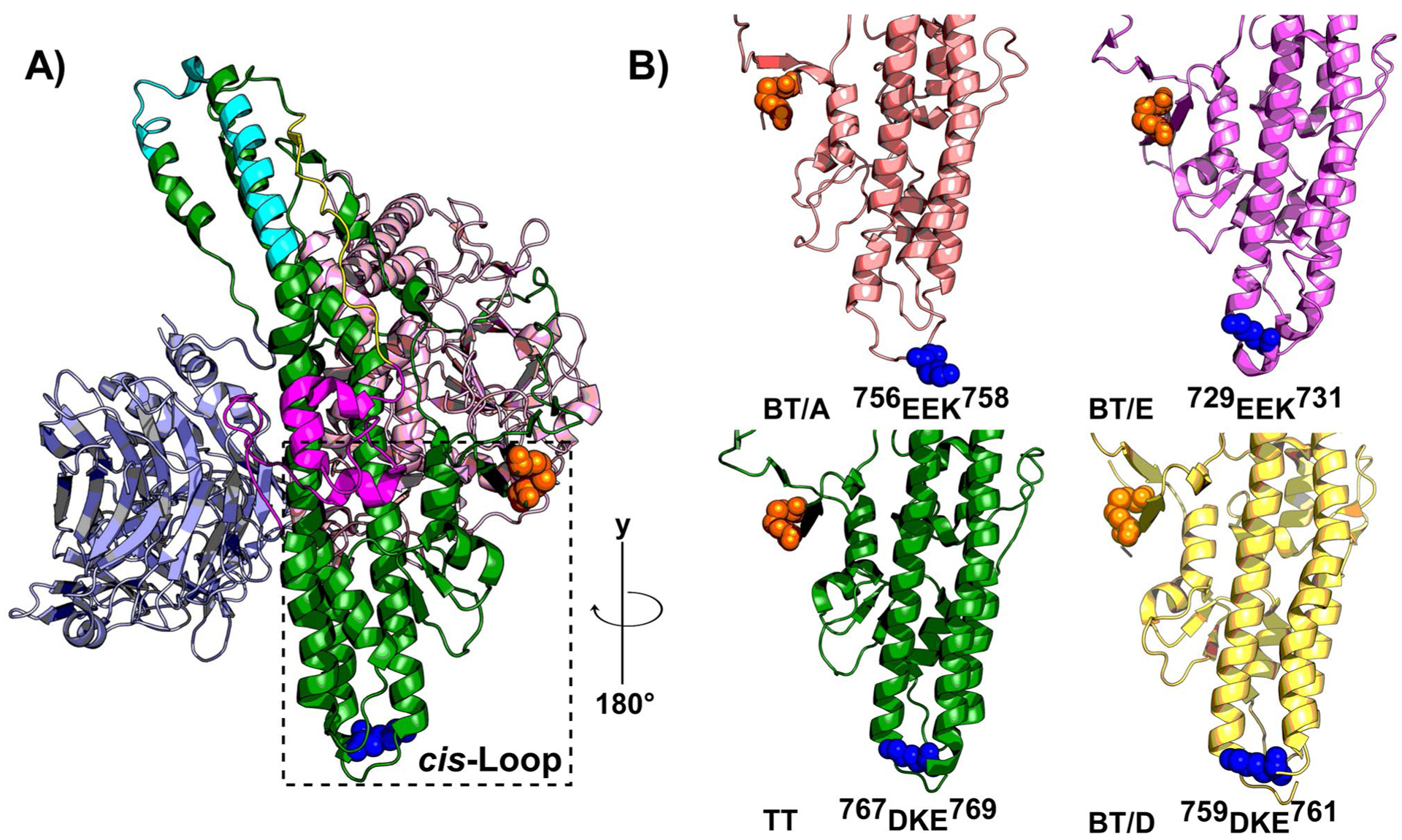
Structural features in Light Chain translocation. **A)** Three domains of tetanus holotoxin (TT) include Light Chain (LC, pink), LC Translocation Domain (HCN, green), and Host Receptor Binding Domain (HCC, light blue). The LC-HC interchain disulfide is shown as orange spheres. Trans-end helices are highlighted in aqua, the membrane penetrating peptide (MPP) in yellow, and the viral fusion peptide (VFP) in magenta. The dashed box denotes the cis-Loop, **B)** BT /A (PDB 3BTA), BT/D (PDB 5BQN) , BT/E (PDB 3FFZ), and TT (PDB 5N0B) cis-Loops are rotated 180 degrees around the y axis of TT in **A)**. The cis-loop sequence, surrounding the conserved lysine, is shown in blue.

Within the CNTs, the HCN is the most conserved and least soluble domain. The limited solubility has presented challenges in identifying distinct residues involved in pore-formation, pH-sensing, and LC translocation. The N terminus of HCN contains a long, unstructured loop known as the “belt”, which wraps around the LC [7]. Downstream of the belt are unstructured loops leading into a pair of 11 nm, kinked helices [7,8]. These long helices are bordered by four shorter helices connected by charged loops. A long, mostly unstructured loop known as the membrane penetrating peptide (MPP) spans across the HCN like a sash and links α12 and kinked helix α14 [7]. The mechanism of LC translocation appears conserved among CNTs and productive LC translocation requires toxin pre-proteolysis, an intact interchain disulfide, and low pH [9]. Features of the HCN previously associated with secondary structures implicated in LC translocation (Table 1) that will be reviewed are shown in Figure 3A. Recently, we developed a novel, cell-based TT-reporter to measure LC translocation potential as the translocation of β-lactamase (β-lac) across a vesicle membrane in live neurons [10].

### Interchain disulfide

The interchain disulfide links LC and HC which physically retains the proximity of LC and HC within the CNT di-chain protein (Figure 3A). Premature reduction of the interchain disulfide reduced toxicity, presumably by loss of proximity of LC and HC preceding LC translocation [11]. In biochemical experiments with CNTs, pH-induced conformational changes were inhibited in pre-reduced toxin, supporting a functional collaboration between LC and HC domains during LC translocation [12]. Pharmacological agents that inhibited the action of thioredoxin-thioredoxin reductase (Trx-TrxR) protected neurons from the action of CNTs, which identified Trx-TrxR as the major system to reduce the interchain disulfide following cytosolic refolding [13,14]. We observed pretreatment of neurons with TrxR inhibitor blocked β-lac translocation, which indicated the movement of the interchain disulfide to the cytosolic face of the synaptic vesicle during LC translocation across the cell membrane [10].

### Belt

The primary amino sequences of the belt among the CNTs are not conserved but contain alternating stretches of hydrophobic and charged residues. Protein modeling suggests that the belt wraps around the LC may act as a pseudosubstrate, while another segment of the belt aligns parallel to the long helical bundles of HCN (Figure 3A) [15]. The belt was proposed to be a chaperone for LC translocation, undergoing rearrangement at low pH to facilitate LC translocation-competency [15], which was supported by studies showing HCN-liposome interactions independent of low pH [16]. In addition, the belt stabilized LC by preventing unfolding in the presence of denaturants such as guanidine chloride, which may prevent premature interaction of the LC with membranes [17]. Electrophysiological experiments showed a beltless HCN/A forms cation-conducting channels in lipid bilayers at either neutral or acidic pH, indicating the belt is dispensable for pore-formation; however other studies have only resolved the LC-HCN as a minimal domain for LC translocation [18]. Future investigations of the belt will determine if the belt has a physiological role during LC translocation across the synaptic vesicle membrane to deliver LC into the cytosol.

### Membrane penetrating peptide (MPP)

Bioinformatics analysis of clostridial sequences identified semi-conserved segments of the HCN with high hydrophobicity including the MPP [19]. An HCN-based peptide, comprising amino acids 659–681 of BT/A (and 669–691 in TT) formed sequence-specific, cation-conducting channels in lipid bilayers analogous to holotoxin [20] (Figure 3A). MPP site-directed spin labeling with LC-HCN/A showed extensive structural rearrangement, but MPP did not interact with membranes at low pH [21]. The role of MPP channel conductance in the holotoxin remains to be resolved.

### BT-switch and viral fusion peptide (VFP)

The BT-switch (residues 620–667 in BT/A) begins upstream of the MPP and is predicted to have an extended conformation or β-strand by bioinformatics [19]. VFP comprises amino acids 634–641 of the BoNT-switch and undergoes conformational changes from loop to β-hairpin under acidic conditions. Introduction of an interchain disulfide lock within the BT-switch of BT/A reduced lipid interaction and toxicity of disulfide locked BT/A [22] (Figure 3A). The disulfide lock within the BT-switch of HCN/A was inefficient in calcein dye release and membrane depolarization measured by ANS fluorescence, indicating BT-switch was involved in conformational changes that functioned prior to pore-formation [22]. Direct assessment of cation-flow across the pore was not performed for the disulfide locked HCN/A or BT/A to assess if this phenotype correlated with loss of channel conductance.

### *Trans*-end helices

The *trans*-end of the holotoxin contains tips of long helices that are physically distanced from the interchain disulfide that connects LC to HC. Protease protection experiments with LC-HCN/A recovered peptides corresponding to the *trans*-end of HCN (residues 805–820) (Figure 3A) and a downstream charged loop of HCN (residues 826–835), indicating the *trans*-end of the holotoxin was protected from digestion at low pH [21]. Peptides were also recovered from regions upstream of the *cis*-Loop (residues 730–744 and 771–778), which may indicate production of a complex intermediate that involves toxin-membrane interaction as various sites within HCN. Subsequent site-directed NBD dye-labeling and site-directed spin-labeling indicate the *trans*-end interacts within a hydrophobic environment, which may initiate early HCN-membrane interactions leading to pore-formation [21].

### *Cis*-Loop

Our TT cell-based reporter assessed two structurally conserved regions near the interchain disulfide. One structure, a charged loop termed the *cis*-Loop, (^767^DKE^769^) is located between α15 and α16 near the interchain disulfide (Figure 3B). Aliphatic mutation of residues in the *cis*-Loop inhibited translocation of LC into the neuronal cytosol, but not pore-formation in Neuro-2A cell membranes [1]. All CNTs contain *cis*-Loops with mixed charges (Figure 3B). While there is no consensus sequence among all serotypes; *cis*-Loop equivalents are found to be similar in HCN of BT(A/B/D/E) and TT crystal structures and each *cis-*Loop equivalent contains an acidic and single conserved lysine residue (Figure 3B). Lysine orientation within the *cis*-Loop is analogous between BT/(B/E/D) and TT crystal structures, with the ε-amino side-chain oriented towards the interchain disulfide, while the longer *cis*-Loop in BT/A oriented away. A similar secondary structural element is also present in diphtheria toxin (DT) (^239^SEEKA^243^); located in the loop between outer helices (αTH2-αTH3), which are also N-terminal to the membrane penetrating helices (αTH8-αTH9) [23].

## Role of the cis-Loop of the translocation domain in LC translocation

### Orientation of the *cis*-Loop during low pH membrane simulation

We performed a course-grain molecular simulation of a beltless HCN/T containing protonation-mimetics of conserved carboxylates to clarify the role of the *cis*-Loop during translocation. The HCN orientation predominantly favored *trans*-end interaction with the lipid bilayer and lasted throughout the simulations [1]. This result is consistent with data that indicates the *trans*-end is protected from digestion at low pH [21]. Our simulation implicates *cis*-Loop orientation away from the bilayer, within a synaptic vesicle the *cis*-Loop could bisect and superficially interact with the inner leaflet. Once the translocon is formed, the *cis*-Loop may facilitate LC translocation. Molecular simulations of DT have implicated known membrane-interacting regions in LC translocation, but have not resolved gross conformational changes during translocation [24]. Therefore, the *trans*end likely represents an early interaction preceding pore-formation, while the *cis*-Loop may act downstream at the site of LC translocation.

### *Cis*-Loop is not the primary pH trigger for LC translocation

Proteins contain a hydrophobic core and present hydrophilic polar or charged residues on their surface to maintain solubility [25]. Many toxins, including DT and the CNTs exploit endosomal acidification; low pH is thought to protonate acidic residues, effectively neutralizing and allowing hydrophobic stretches to interact with the membrane [25]. Among the CNTs, HCNs contain six conserved and two semi-conserved carboxylate residues, and most localize to the toxin face containing the interchain disulfide and viral fusion peptide. One LC and two HCN carboxylates ^653^E and ^877^D were mutated in BT/B to mimic protonation [26]. These mutations resulted in faster onset of paralysis and pH-independent substrate cleavage in cells treated with an endosomal acidification inhibitor, bafilomycin A1 [26]. The TT *cis*-Loop contains two soluble carboxylates, one semi-conserved at ^767^D. Charge reversal of the *cis*-Loop (^767^RKK^769^) did not uncouple pH-sensitive translocation of the TT-reporter, indicating acidic residues of the *cis*-Loop are not major drivers of pH-induced conformational changes [1].

### *Ci*s-Loop is independent of pore-formation

Mutations that reduce toxin potency may map to pore-forming regions of toxins, as reported in the double dagger catalytic domain translocation models of DT and Tcdb [23,27,28]. These mutations prevent membrane insertion and inhibit channel-conductance or liposome release assays. The TT reporter *cis*-Loop aliphatic variant (^767^AAA^769^) formed pores large enough for trypan uptake in neuronal membranes, but arrested LC translocation at a nonproductive intermediate [1]. Our data cannot resolve if the LC translocon of TT(^767^AAA^769^) is in a pre-pore state that cannot accommodate LC translocation.

Current models hypothesize that the long helices may break and insert to form the translocon, which is supported by data that indicates secondary regions upstream from the *cis*-Loop (residues 756–758 in BT/A) are protease protected [21]. Indeed, mutations in the pore-forming subunits of anthrax toxin can prevent conversion of a pre-pore into a mature pore, uncoupling peptide translocation [29,30]. Similarly, carboxylate charge reversals in anchoring loops between transmembrane regions result in translocation inhibition for DT and *C. difficile* toxin B, a cytotoxin produced by *C. difficile,* TcdB [27,31]. Since the TT(^767^RKK^769^) forms pores, the *cis*-Loop is unlikely to be directly penetrating the membrane.

### *Cis*-Loop variant LC-HCN(^767^AAA^769^) does not cleave VAMP2

Incubation of catalytically active LC-HCN(^767^AAA^769^) with primary neuron cultures confirmed translocation inhibition correlated with reduction of VAMP2 cleavage, the substrate of TT [1]. Taken together, mutation of the cis-Loop inhibited LC cytosolic delivery in neuronal cells, indicating a distinct role during translocation (MZ, unpublished data).

## Overview of cis-Loop Potential Mechanisms of Action

### CNT LC delivery in neurons

In addition to exploiting host receptors, endosome maturation, and breaching membranes, CNTs use host factors for refolding and trafficking to the target substrate. BT and TT exploit host chaperones including Trx and heat shock protein 90 (Hsp90) for interchain disulfide reduction and refolding, respectively. Similarly, DT uses Trx, Hsp70, and COPI complex for interchain disulfide reduction, refolding, and escaping the endosome [32,33]. Translocated catalytic domains mimic nascent peptides and are refolded upon entry into the cytosol; however, consensus sequences have not been identified for CNT HCN or LC-chaperone interactions.

The *cis*-Loop contains a carboxylate and a conserved lysine located in a secondary structural element. Prediction of function based on structural motif alone can be problematic, but valuable in formulating testable hypotheses. Many proteins have conserved secondary structures yet perform widely different functions. For example, this feature is observed in mammalian proteins that bind acidic DNA and calcium binding proteins [34]. The *cis*-Loop requires a positive charge on a loop to facilitate LC translocation, indicating charge plays a role in a downstream interaction potentially with acidic lipids, the HCN, or LC. We will discuss other proteins containing *cis*-Loop structures to propose how the *cis*-Loop may function during translocation.

### Eukaryotic J proteins

The *cis*-Loop may act as a LC chaperone following pore-formation and initiation of LC translocation. As peptides upstream from the *cis*-Loop were found to be protease-protected, the *cis*-Loop may be re-oriented near or within the translocon, proximal to the interchain disulfide and LC C terminus. The *cis*-Loop resembles the J-domain in Hsp40, a diverse family of eukaryotic proteins that are obligate partners of Hsp70. The J-protein family proteins contain a charged motif (HPD) on a loop and function to bind nascent peptides, stimulate ATPase activity in Hsp70, and present extended peptides for translocation across membranes [35–37]. In yeast, mitochondrial J-proteins Tim14 and Tim16 contain the canonical HPD sequence and a DKE variant, respectively, that associate to stabilize the TIM23 translocase, which includes Hsp70 [37]. By structural analogy, the TT *cis*-Loop may facilitate interaction of an unfolded LC into the HCN translocon alone or through interactions with an Hsp70 protein. J-proteins have a similar requirement of charge for their co-chaperone activities, with aliphatic substitution having lethal phenotypes in yeast [35]. The activities of Hsp40/Hsp70 initiate translocation and folding, but Hsp90 is shown to cooperate downstream [38]. If the *cis*-Loop functions in LC peptide translocation, we speculate the *cis*-Loop does not have additional activities of Hsp40, but rather provides an anchor for LC translocation into the HCN pore, analogous to the anchoring properties of Hsp40 with Hsp70. Once the LC interchain disulfide translocates through the pore, Trx reduces the bond and the nascent peptide is refolded by Hsp90 [32].

### Alternative functions of the *cis*-Loop

The CNT translocon is unresolved and the HCN may undergo additional conformational changes that are not revealed by changes in secondary structure. Following acidification, the *cis*-Loop may be more favorably positioned to facilitate protein interactions. We predict these interactions may be electrostatic or due to formation of a salt-bridge that directs and stabilizes the LC within the translocon. The *cis*-Loop is not required for pore-formation but is proximal to the interchain disulfide (42.5 nm between Cys^466^ and Lys^768^ in the TT crystal structure, PDB:5N0B) and below the VFP. Interaction between the *cis*-Loop and the unstructured C terminus of the LC could initiate C- to N- terminal translocation.

More recent translocation data and models support formation of dimers or trimers pre-requisite to translocation. The neuronal lipid-binding protein alpha synuclein contains charged repeats of KTKEGV, within loops and helices. These charged repeats facilitate interaction with phospholipids within the inner membrane [39]. In addition, the charges are required to promote oligomerization of the alpha synuclein tetramer [39]. Thus, *cis*-Loop may facilitate direct inter-molecular interactions between toxins leading to LC translocation. *Cis*-Loop could also mediate electrostatic interactions with acidic lipids in the endosome to promote LC translocation with or without oligomerization.

### Sensors of curvature

*Cis*-Loop and *trans*-end may act in concert on the HCN as sensors of curvature to promote translocation competency. Helical proteins, including gp41 and colicin family members sense curvature of lipid faces and induce fusion [40–42]. The mammalian BAR family, composed of a dimer of helical bundles with charged ends, sense and induce membrane curvature. Most BAR domains act on the positive curvature of a vesicle (cytosolic side of vesicle), but some family members recognize negative curvature [43]. HCN can form pores and cation-conducting channels in lipid bilayers, but under physiological conditions, may sense pH and curvature inside the endosome to trigger pore-formation.

### *Cis*-Loop in proposed models

Two models proposed for CNT translocation are the tunnel and cleft model [25]. The tunnel model is a protein channel, like voltage-gated channels, and is pre-requisite for translocation to occur. Most support for this model comes from electrophysiological experiments measuring cation-conductance [44]. Pore-forming translocons are considered analogous with cation-conducting channels; however, recent mutations found to uncouple diphtheria toxin channel conductance did not affect translocation [45]. For CNTs, it is not resolved whether the role of conductance is an intermediate leading to pore-formation or a separate, parallel interaction. Many protein toxin channels in lipid membranes conduct ions, but not larger molecules such as sugars or amino acids [25]. The overall conductance of these channels is also quantitatively lower than mammalian translocons found in the ER [25]. The HCN channel is estimated to accommodate a single alpha helix; however, this does not explain the translocation of the intact interchain disulfide to the cytosol prior to translocation, as the interchain disulfide connects the LC and HCN [10,31]. Our past investigations and action of Trx inhibitors indicate interchain disulfide reduction is necessary to facilitate LC translocation and toxicity.

The cleft model allows for deformation of a bilayer and ratcheting through peptides which contact both lipids and protein side-chains [25]. The cleft model supports translocation of larger secondary structures and data indicates HCN and LC domains are pH stable and do not exhibit large structural changes required for LC unfolding. Circular dichroism of these domains reveals that the LC undergoes structural rearrangement from α-helical to β-sheet at low pH [46]. Current translocation secondary structures including the VFP, the protease-protected regions, and the *cis*-Loop may be supported by either model at the field’s current resolution. If pore-formation and LC delivery are linked, as observed for other toxins, abrogation of pore-formation should correlate with cytotoxicity [28]. More work is required to identify the minimal HCN domain in LC delivery. In addition, investigations should focus on LC participation during translocation and pore-formation in cells, as catalytic subunits have been demonstrated to engage in membrane-interactions [17,21,47].

### *cis*-Loop utility

The recent discoveries of BT/Pmp1, BT/En, and BT/Wo confirm that the BT toxin reservoir is more diverse than previous assumptions [2]. The development of a pan-vaccine against BTs has been an overwhelming task. Most strategies have focused on soluble subunit vaccines such as the HCC, but holotoxins as immunogens have identified neutralizing epitopes in all three domains [48]. Until recently, less emphasis was placed on targeting the antigenic regions of the HCN, which has the potential to neutralize multiple serotypes due to high conservation [49]. Translocation is a rate-limiting step in the mechanism of intoxication and HCN is the most conserved domain [9]. Our past studies have identified two critical regions that supported LC translocation, the intact interchain disulfide and the *cis*-Loop that is structurally conserved among TT, BT, and clostridial-like neurotoxins [1,10]. The BT-switch represents another conserved secondary structural element that supports translocation processes [22].

Two studies demonstrated neutralization potential by targeting these secondary structural elements. The single-chain fragment, 4E17.1, identified by yeast-display, bound the *cis*-Loop in BT/A with residues Y^750^, Y^753^, E^756^, and E^757^ critical for interaction (D^767^ in TT) [50]. This antibody recognized BT/(A,B,E,F), the causal serotypes of human botulism and may recognize additional serotypes. More recently, the camelid antibody ciA-B5 was found to bind near the BT-switch and prevent calcein release from liposomes [51]. Rational design of immunogens containing these structures can be used to stimulate broadly neutralizing antibodies in individuals against CNTs. This strategy is currently implemented against influenza hemagglutinins and gp41, both conserved, helical proteins involved in the viral membrane fusion of influenza and HIV, respectively [52,53]. Identification of neutralizing antibodies can lead to production of antitoxins for prophylactic administration upon toxin exposure to minimize degree or duration of paralysis. Antibodies that bind the HCN may sterically inhibit toxin association with receptors or distinct intermediates in translocation such as structural rearrangement, pore-formation, or LC delivery. Additionally, design of immunogens that stabilize secondary structural elements or inhibit toxin action without chemical-inactivation will improve current vaccines [54]. Last of all, the screening of current drugs and small molecule libraries may reveal compounds that prevent HCN secondary structure interaction with the membrane or downstream host chaperones.

## Closing Statement

Continued extrapolation of the molecular and structural basis for LC translocation may provide additional applications of BT as a human therapy or alternatively target CNTs for neutralization to mediate disease by these most toxic human proteins.

## Figures and Tables

**Table 1:** Clostridial neurotoxin HCN secondary structures implicated in LC translocation^a^.

name	sequence and residues	secondary structure [7]	proposed role	therapeutic potential
**Interchain disulfide**	**C**^**439**^**-C**^**466**^ (TT)	disulfide between LC and HCN domains	structural linkage and reduction facilitates LC translocation [10, 12]	inhibitors ebselen and PX-12 (Trx), auranofin and curcumin (TrxR) [14]
**belt**	^492^**ENISLDLIQQYYLTFNFDNEPENISIENLSSDIIGQLELMPNIERFPNGKKYEL**^**545**^ (BT/A)	predominantly unstructured loop with short helices	LC chaperone that regulates membrane interaction [18]	monoclonal antibodies N5 and N6 [55, 56]
**Membrane penetrating peptide**	^659^**GAVILLEFIPEIAIPVLGTFALV**^**681**^ (BT/A) ^669^**GVVLLLEYIPEITLPVIAALSIA**^**691**^ (TT)	loop containing helices α13 and η11, and strand β19	structural rearrangement and ion channel formation[20]	none identified
**BT-switch/_****viral fusion peptide**	^620^**EVSTTDKIADITIIIPYIGPALNIGNMLYKDDFVGALIFSGAVILLEF**^**667**^ (BT/A)	loop containing helices η10 and α13	structural rearrangement before membrane penetration [22]	camelid antibody, ciA-B5 [51]
***trans-*end helices**	^8o5^**VKRLEDFDASLKDALL**^**820**^ ^826^**NRGTLIGQVD**^**835**^ (BT/A)	helices α17 and α18	membrane penetration [21]	monoclonal antibody N26 [56]
***cis*-Loop**	^767^**DKE**^**769**^ (TT)	loop between α15 and α16	LC chaperone that anchors at translocon [1]	single-chain variable fragment antibody, 4E17.1 [50]

a Residues shown in red are conserved and purple are semi-conserved among CNTs serotypes [8]. Residues assigned based on CNT used in study
